# Dietary Intake of Young Male Ice Hockey Players 10–13 Years of Age during a Week-Long Hockey Camp

**DOI:** 10.3390/nu14142978

**Published:** 2022-07-21

**Authors:** Tyler F. Vermeulen, Stephanie M. Boville, Alexander S. D. Gamble, Jessica L. Bigg, Devin G. McCarthy, Gregory S. Eskedjian, Lawrence L. Spriet

**Affiliations:** Department of Human Health and Nutritional Sciences, University of Guelph, Guelph, ON N1G 2W1, Canada; stephaniemboville@gmail.com (S.M.B.); alexander.gamble@guelphhumber.ca (A.S.D.G.); jbigg@uoguelph.ca (J.L.B.); mccartdg@mcmaster.ca (D.G.M.); g.eskedjian@gmail.com (G.S.E.); lspriet@uoguelph.ca (L.L.S.)

**Keywords:** nutrition, children, adolescents, ice hockey, energy intake, macronutrients, micronutrients

## Abstract

This study recorded the dietary intakes of young male ice hockey players (10–13 year (yr)) for 3 consecutive days while participating in a 5-day summer hockey camp. Players were categorized as older children (OC, *n* = 10; 10.7 ± 0.2 yr; 37.1 ± 1.5 kg; 147.9 ± 2.1 cm) and young adolescents (YA, *n* = 10; 12.9 ± 0.1 yr; 45.2 ± 1.5 kg; 157.0 ± 2.4 cm). Players consumed their usual daily intakes. Parents recorded food intake in the mornings and evenings, while the researchers recorded food intake at camp. Energy intake was higher in both groups when compared to data for age-matched young Canadian (CDN) males (OC, 2967 ± 211 vs. 2000 kcal/day; YA, 2773 ± 91 vs. 2250 kcal/day). Carbohydrate (CHO) (OC, 11.2 ± 0.8 vs. YO, 8.9 ± 0.5 g/kg body mass/day) and protein (OC, 3.2 ± 0.3; YO, 2.4 ± 0.1 g/kg/day) intakes were higher than reported for young CDN males (CHO, 3.6 and protein, 1.0 g/kg/day) and were within the Acceptable Macronutrient Distribution Range (AMDR; CHO, 56 ± 2.3; 57.4 ± 0.8%; protein, 16.1 ± 1.0; 15.7 ± 0.7%). Fat intake was also within the AMDR in both groups (OC, 29.8 ± 1.6%; YA, 28.3 ± 1.0%). Micronutrient intake was adequate except for Vitamin D intakes that were below the recommended 15 ug/day at 6.3 ± 0.7 (OC) and 5.0 ± 1.5 ug/day (YA). In summary, energy and macronutrient intakes of the OC (10–11 yr) and YA (12–13 yr) players were high and well above the age matched CDN norms. The older children had higher energy intakes/kg body mass than the young adolescents. Higher energy intakes allowed for micronutrients intakes to be met in these young active males, except for vitamin D intake.

## 1. Introduction

Ice hockey is a sport that is characterized by explosive bursts of all-out effort interspersed with shorter periods of less intense activity or rest [1,2]. The physiological demands can be high when playing at an elite level and require increased cardiovascular capacity, muscular endurance, speed, strength, power, flexibility, and agility. Training has become a year-round endeavor, typically involving 3 to 4 training phases to ensure that players maintain their fitness level and training focus [2].

Carbohydrate has been shown to be the major fuel for ice hockey in order to complete high intensity aerobic activities, such as skating, and anaerobic activities such as sprinting and grappling [3,4,5]. Specifically, adequate muscle glycogen is required to maintain performance and offset fatigue while playing this intermittent, stop-and-go sport [6,7]. However, few researchers have examined the energy and nutritional intake of hockey players and these studies examined adult male and female players [8,9,10]. While Reading et al. [11] examined the nutritional knowledge and education of adolescent male hockey players, and Parnell et al. [12] examined the energy intake of 11–13 year (yr) male athletes, we are unaware of any research that has examined the dietary energy and nutritional intake of young male hockey players aged 10–13 years.

The intake of sufficient energy, timing of meals, and proper hydration are areas of focus that the young athlete needs to consider when formulating a training regime [13,14,15,16,17]. Adolescent athletes are a unique population, since they not only need fuel for daily athletic events but also need to consume a well-balanced diet to ensure optimal growth and health [13,15]. Sports nutrition is becoming more prominent in all facets of the sporting world [18] and ice hockey players are becoming more interested in learning about nutrition and how it relates to their performance outcomes.

It is assumed that nutritional needs would be increased for elite-level hockey players in comparison to age-matched Canadian (CDN) youth and, therefore, exceed the recommendations set forth by Health Canada [19] for energy and macronutrient intakes of healthy youth. The young hockey players would be expending large amounts of energy with on-ice and off-ice training and on-ice games almost every day during the season. Increases in macronutrient consumption would be required to obtain sufficient energy to fuel the activities performed by each player. Therefore, the purpose of this study was to measure dietary intake over a 3-day period during a 5-day hockey camp in young (10–13 yr), healthy ice hockey players. We hypothesized that they would consume more energy and macronutrients than recommended for CDN youths and consume the recommended micronutrient intakes.

## 2. Materials and Methods

### 2.1. Participants

This study involved 20 young male ice hockey players who attended a week-long summer hockey camp. Groups were separated into older children (OC; *n* = 10; 10.7 ± 0.2 yr) and young adolescents (YA; *n* = 10; 12–13 yr) based on an age division from Health Canada (2010). Other characteristics are in Table 1. To our knowledge, all participants and parents had no previous experience completing a 3-d dietary record. Prior to receiving consent, all participants and parents were informed both orally and in writing of the study procedures, requirements, and possible risks associated with the study. The participants and parents provided written and oral consent for inclusion and the study was approved by The Research Ethics Board of the University of Guelph.

### 2.2. Study Design

Participants completed a dietary record on 3 consecutive days (Tuesday–Thursday) during a week-long summer hockey camp. They were instructed to maintain their normal eating patterns for the duration of the study. Prior to recording, all parents met with a researcher and were instructed on how to measure and record all food items. Relevant instructions and examples, in the form of food pictures with sizing portion and a template food intake sample, were provided to the parents for their use. The parents of the participants were asked to record food intake in the mornings before the camp and in the evening for the dinner meal and snacks after the camp, while the researchers recorded all food and fluid intake during the daytime at camp. The researchers met with each athlete during their lunch break to observe and record lunches. Parents met with the researchers daily to check on adherence of the dietary recordings and answer any questions. Food records were analyzed using ESHA nutritional software (ESHA Processor Nutrition Analysis Software, Salem, OR, USA) and data were inputted by two researchers previously trained with this software. All data entries were checked and verified by both.

### 2.3. Hockey Camp Exercise Protocol

The camp was designed to focus on combining skill development with high-intensity interval training. There were five hours of on-ice and one hour of off-ice training per day. On-ice drills included skating, endurance, acceleration, puck handling, and shooting skills. Off-ice training involved strength and circuit training, aerobic conditioning, flexibility, and stretching.

### 2.4. Comparison with Canadian Nutritional Recommendations for Youth

The data from this study were compared to the recommended energy and macronutrient intakes from the 2010 Health Canada Daily Recommended Intake (DRI) report. Macronutrient intakes were referenced as a percent of total energy intake of Acceptable Macronutrient Distribution Range (AMDR) and absolute weight.

### 2.5. Statistical Analysis

All data are reported as means and standard error of the means (SEM). No statistics were used to compare measured dietary intake results with recommended amounts. Unpaired *t*-tests were used to determine if differences existed between the OC and YA groups. Results were considered significant when *p* ≤ 0.05.

## 3. Results

### 3.1. Energy Intake

The absolute daily energy intake was higher during the hockey camp (OC, 2967 ± 211; YA, 2773 ± 91 kcal/day) compared to the average energy intake for age matched CDN youth (OC, 2000; YA, 2250 kcal/day) (Table 2). Moreover, the absolute energy intake/kg body mass (BM) was higher for the OC group compared to the age-matched population of CDN youth (81 ± 5 vs. 56 kcal/kg BM/day) and the YA group (Table 2). However, the YA group energy intake expressed per kg BM was similar to CDN recommendations (62 ± 3 vs. 63 kcal/kg/BM/day).

### 3.2. Macronutrient Consumptions

In accordance with the 2010 Health Canada DRI report, it is recommended that a person between the ages of 4 and 18 years consume 45–65% carbohydrate, 10–30% protein, and 25–35% of fat during each day. These ranges are better known as the AMDR. The OC (58 ± 2% CHO, 16 ± 1% protein, and 30 ± 2% fat intakes/day) and YA (57 ± 1% CHO, 16 ± 1% protein, and 28 ± 1% fat intakes/day) groups both fell within the recommendations of the AMDRs.

### 3.3. Carbohydrates

The recommendation for carbohydrate intake is 130 g/day or 3.6 g/kg/day for Canadian youth 9–13 years of age, weighing 36 kg, and 163 g/day or 4.5 g/kg/day for youth in this age group weighing 45 kg. The total CHO intake per day and the amount relative to body mass were higher in both groups compared to the CDN recommendations (Table 2). The OC CHO intake was significantly higher than the YA intake on a BM basis (11.2 ± 0.8 vs. 8.9 ± 0.5 g/kg/day).

### 3.4. Protein

Both groups of young hockey players consumed more protein on a total and per kg BM basis than the CDN recommendations suggest (Table 2). The daily protein consumption was similar on a BM basis between the two groups, but the OC group consumed significantly more protein on a BM basis than the YA group (3.2 ± 0.3 vs. 2.4 ± 0.1 g/kg/day).

### 3.5. Fat

There are no specific recommendations for total fat intake for these age groups. Total fat consumption was similar between groups and the OC group (2.7 ± 0.3 g/kg/day) consumed significantly more fat/kg BM than the YA (1.9 ± 0.1 g/kg/day) group (Table 2).

### 3.6. Water/Fluid

Both groups of players consumed more fluid than age matched CDN youth. The water intake was similar between the OC group (2.8 ± 0.4 L) and the YA (3.1 ± 1.2 L).

### 3.7. Vitamins

The recommended intake for vitamins was met for both groups except for Vitamin D (Table 3). Both the OC and YA groups were under the CDN recommendations for vitamin D of 15 μg/day and had intakes of 6.3 ± 0.7 and 5.0 ± 1.5 μg/day, respectively. Greater absolute amounts of vitamins A, C, E, K, B2, B12, and niacin were reported for the YA vs. OC players (Table 3).

### 3.8. Minerals

The recommended intake of minerals was met for both groups, except for slightly low values for calcium and phosphorous in the YA group (Table 3). The sodium intakes were much higher than recommended in both groups, but this would relate to replenishing the large amount of NaCl lost while sweating during the hockey camp. Interestingly, while meeting the mineral requirements, YA players consumed significantly less calcium, iron, phosphorous, and zinc than OC players (Table 3).

## 4. Discussion

All but one participant had a BMI (kg/m^2^) within the healthy percentile range indicating good fitness levels. A healthy BMI for these young athletes can be achieved by having a high physical activity, high sleep, and low sedentary behavior in comparison to an unhealthier state of having low physical activity, low sleep, and high sedentary behavior [20]. Saunders et al. [21] suggested that health benefits will arise when moderate-to-vigorous-intensity physical activity (MVPA) is used in replacement of sedentary behavior. The number of OC and YA age-matched youths in the general Canadian population engaged in MVPA was only 4%, while 18% participated in light-intensity physical activity, according to the Canadian Health Measures Survey [20]. During the week-long ice hockey camp, the participants in this study were involved in high-intensity physical activity for approximately six hours/day. The camp was designed to prepare the participants for the upcoming season by subjecting them to vigorous activities to improve their fitness and conditioning levels. As we hypothesized, the large energy demands of the hockey camp, required energy intakes that were much higher that recommended for average CDN youth and this study reported energy intakes that were 48% and 23% higher than these recommendations. With these large energy intakes, macronutrient recommendations were exceeded and most recommendations for vitamins and minerals were also met in both groups, with the one major exception being vitamin D.

### 4.1. Macronutrient Consumption

The absolute macronutrient intakes of both the OC and YA groups were much higher than recommendations. These macronutrient intakes were needed to fuel (CHO) the intense physical activity that was undertaken and in recovery (protein). The data revealed that both groups of young males had CHO, protein, and fat macronutrient intakes that fell within the AMDR ranges (45–65% CHO, 25–35% fat, and 10–30% protein).

#### 4.1.1. Carbohydrate

CHO intakes were 215% and 145% higher than recommendations for CDN youth. CHO are the preferred fuel source during high-intensity training and stop-and-go competitions, with ice hockey being no exception [3,6,7]. CHO is metabolized to produce both aerobic and anaerobic energy for high intensity aerobic and sprinting demands. Ice hockey demands a combination of both energy systems and thus requires large amounts of CHO to fuel both.

An important aspect of CHO storage is via muscle and liver glycogen stores. A joint position statement from the American Dietetic Association, along with the Dietitians of Canada [22] suggested that consumption of 500–600 g/day (or 7–8 g/kg BM/day for a 70-kg individual) is required in the maintenance of CHO stores in the muscle and liver during training and competition. However, these amounts are relevant for adult endurance athletes specifically. The OC and YA groups in this study both had CHO intakes above the recommendations of 7–8 g/kg/day. The OC had the greatest intake at 11.2 ± 0.8 g/kg, compared to the YA intake of 8.9 ± 0.5 g/kg. Our results matched quite closely with results in a group of 11–13-year-old male athletes where CHO intake was 8.1 g/kg BM estimated from a 24 h recall with a Food Behaviour Questionnaire [12]. These large intakes are attributed to the increased total energy expenditure by these young and very active males, plus the need for growth energy.

#### 4.1.2. Protein

Protein is needed to maintain skeletal muscle function in young individuals. It plays a significant role in recovery [3] and the current DRI for all healthy individuals within these ages is between 0.95–1.2 g protein/kg/day. Aerenhouts et al. [23] and Desbrow et al. [24] suggested that total daily protein intake of ~1.5 g/kg/day (e.g., ~0.3 g/kg × 5 equally spaced meals/snacks) is required to replace amino acid oxidation incurred from activity and training, and to also maintain whole body net protein balance, and support the normal growth and development of adolescent aged athletes. The OC and YA groups had consumptions greater than the recommendations at 3.2 ± 0.3 and 2.4 ± 0.1 g/kg and were 250% and 150% higher than recommendations. Data from Parnell et al. [12] reported a similar protein intake value of 2.4 g/kg BM in 26 young 11–13 yr male athletes.

#### 4.1.3. Fat

There are no specific recommendations for fat intake after the first year of life [19]. Fat is beneficial for transport of fat-soluble vitamins, hormone production, and a virtually unlimited supply of energy for lower exercise intensities. Youths within the ages of 10–13 are advised to consume 25–35% of their daily intake from healthy fats, including polyunsaturated and monounsaturated fats and both the OC and YA groups achieved these percentages in the present study.

### 4.2. Micronutrient Consumption

Health Canada [19] reported that Canadian youth of 9–18 years of age had adequate intakes of most micronutrients with the exceptions of vitamins A and D, calcium, and potassium. In the present study, most vitamins and minerals were adequately consumed or were within ~10% of the recommended intake. However, dietary intakes alone can be insufficient when determining nutritional status as adequate or optimal and should be accompanied by blood biomarkers to verify. The higher need for energy intake when very physically active aided in reaching and surpassing these requirements.

Vitamin D intake was the one exception where intakes were less than half the recommend levels. Parnell et al. [12] also reported low Vitamin D intake of only 56% of recommended amount in their cohort of young active males. Vitamin D is essential for the bone growth and mineralization in young, developing children and adolescents [25]. It can also factor into autoimmunity and metabolic function [14,25]. It should be noted that the majority of vitamin D is synthesized from the sun light (UV radiation)—especially during the summer months in Canada—with sun exposure on exposed areas of 5–15 min around midday (10 am–2 pm), several times per week being recommended. Dietary sources are low in vitamin D content and blood measures would be needed to determine if these low dietary intakes translate into actual deficiencies in the body [26]. It has been reported by Health Canada [19] that vitamin D intake is inadequate from dietary sources for most Canadian adolescents (at 84.5%), and the present group were no different. None of the OC group had levels close to the recommendations (15 μg/day), while only one individual of the YA group was at that level (14.9 μg/day).

Iron is needed for oxygen delivery to tissues. It also supports the increasing blood volume and lean muscle mass during growth and development [15]. Recommendations were met by all participants in each group with the OC consuming ~26 mg/day and the YA consuming ~19 mg/day. These intakes were more than doubled the recommendation of 8 mg/day. Each participant ate similar foods each day of recording, but choose a variety of heme- and non-heme-containing foods that included beef, poultry, pork for heme-rich sources, and fortified cereals and breads for non-heme sources.

The Health Canada DRI report [19] found sodium intakes to be in excess of the upper limit (UL) recommendations in CDN youth and the present results found the same high sodium intake. Sodium is an important electrolyte in the body and is required for nerve transmission and fluid retention. Adverse health effects of sodium include increased blood pressure and hypertension. However, large amounts of sodium can be lost through sweat. Previous work reported that large amounts of sodium were lost (~4200–4400 mg/day) in sweat throughout a day in young male athletes involved in physical exertion [27]. The present study reported sodium intakes of ~4300–4400 mg/day and Parnell et al. [12] also reported a high sodium intake of 3476 mg/day in 10–13-year-old male athletes. A potential concern is that the intakes found in both the OC and YA groups were more than double the UL recommendation of 2200 mg/day. However, no guidelines are available for active children that lose large amounts of sodium in sweat, so the UL value does not seem relevant for young athletes [12,27].

## 5. Conclusions

Young active athletes require high levels of energy to fuel their physical activity and for continued growth. As hypothesized, older children (10–11 yr) and young adolescent (12–13 yr) male ice hockey players had high levels of energy and macronutrient intakes to fuel their movements during a hockey camp requiring 6 h of physical activity/day for 5 consecutive days. CHO and protein intakes were very high at ~9–11 and 2.4–3.2 g/kg body mass/day. Micronutrient intakes were above recommendations for age-matched young males except for vitamin D, which was about one-third of the recommended intake.

## Figures and Tables

**Table 1:** Characteristics of the older children (OC) and young adolescent (YA) ice hockey players.

Characteristics	OC, 10–11 Yr (*n* = 10)	YA, 12–13 Yr (*n* = 10)
Age (years)	10.7 ± 0.2 [10–11]	12.9 ± 0.1 [12–13] *
Body Mass (kg)	37.1 ± 1.5 [30.0–45.4]	45.2 ± 1.5 [35.0–52.3] *
Body Mass Index (kg/m^2^)	16.9 ± 0.6 [15.5–20.5]	18.3 ± 0.4 [17.3–21.1]

Values are ± SEM with range in square brackets. * Significant difference between OC and YA (*p* < 0.05).

**Table 2:** Absolute and relative daily dietary intakes for energy and macronutrients in older children (OC) and young adolescent (YA) players compared to the recommended amounts for Canadian age-matched young males.

	Recommendations (10–11 Yr)	OC, 10–11 Yr (*n* = 10)	Recommendations (12–13 Yr)	YA, 12–13 Yr (*n* = 10)
Energy (kcal)	2000	2967 ± 211 (10/10)	2250	2773 ± 91 (9/10)
Energy (kcal/kg)	56	81 ± 5 (10/10)	63	62 ± 3 * (4/10)
Carbohydrate (g)	130	410 ± 27 (10/10)	163	399 ± 16 (10/10)
Carbohydrate (g/kg)	3.6	11.2 ± 0.8 (10/10)	4.5	8.9 ± 0.5 * (10/10)
Carbohydrate (%)	46–65	56 ± 2 (9/10)	46–65	57 ± 1 (10/10)
Fat (g)	ND	100 ± 10	ND	87 ± 4
Fat (g/kg)	ND	2.7 ± 0.3	ND	1.9 ± 0.1 *
Fat (%)	25–35	30 ± 2 (8/10)	25–35	28 ± 1 (10/10)
Protein (g)	34	120 ±12 (10/10)	43	108 ± 5 (10/10)
Protein (g/kg)	0.95	3.2 ± 0.3 (10/10)	1.20	2.4 ± 0.1 * (10/10)
Protein (%)	10–30	16 ± 1 (10/10)	10–30	16 ± 1 (10/10)
Water (L)	2.4	2.8 ± 0.4 (5/10)	2.4	3.1 ± 1.2 (10/10)

Mean values ± SEM. * Significant difference between OC and YA (*p* < 0.05). Recommendations based on 36 and 45-kg reference weights for both age groups (Health Canada DRI report, 2010). ND, no data provided by Health Canada report. Numbers in parentheses indicates how many participants met the recommendations in their groups.

**Table 3:** Older children (OC) and young adolescent (YA) player’s daily intake of water, fiber, vitamins, and minerals compared to age recommended values.

Nutrient	Recommendations 9–13 Yr	OC, 10–11 Yr (*n* = 10)	YA, 12–13 Yr (*n* = 10)
Vitamin A (mcg RAE)	600	833 ± 77 (9/10)	674 ± 62 * (5/10)
Vitamin D (μg)	15	6 ± 1 (0/10)	5 ± 2 (1/10)
Vitamin E (mg)	11	4 ± 1 (0/10)	8 ± 2 * (2/10)
Vitamin K (mcg)	60	35 ± 7 (1/10)	71 ± 14 * (6/10)
Vitamin B1 (mg)	0.9	2.1 ± 0.2 (10/10)	1.6 ± 0.3 (9/10)
Vitamin B2 (mg)	0.9	3.0 ± 0.3 (10/10)	1.8 ± 0.3 * (8/10)
Vitamin B6 (mg)	1.0	2.5 ± 0.3 (9/10)	2.1 ± 0.4 (9/10)
Vitamin B12 (μg)	1.8	11.4 ± 1.8 (10/10)	5.6 ± 1.2 * (9/10)
Vitamin C (mg)	45	120 ± 23 (9/10)	187 ± 25 * (10/10)
Niacin (mg)	12	28 ± 2 (10/10)	23 ± 3 * (9/10)
Folate (μg)	300	452 ± 75 (8/10)	414 ± 81 (6/10)
Calcium (mg)	1300	1664 ± 158 (6/10)	1252 ± 137 * (3/10)
Magnesium (mg)	240	282 ± 24 (7/10)	274 ± 47 (4/10)
Iron (mg)	8	26 ± 2 (10/10)	19 ± 2 * (10/10)
Phosphorous (mg)	1250	1555 ± 175 (6/10)	1091 ± 133 * (4/10)
Potassium (mg)	2500	3048 ± 280 (7/10)	2757 ± 351 (6/10)
Zinc (mg)	8	15 ± 2 (9/10)	10 ± 2 * (5/10)
Sodium (mg)	1500	4296 ± 438 (10/10)	4435 ± 317 (10/10)

Mean values ± SEM. * Significant difference between OC and YA (*p* < 0.05). Recommendations are dietary reference intakes from Health Canada (2010). RAE = retinol activity equivalents. Numbers in parentheses indicates how many participants met the recommendations in their groups.

## Data Availability

Data is contained within the article.
